# New Insights in Trigeminal Anatomy: A Double Orofacial Tract for Nociceptive Input

**DOI:** 10.3389/fnana.2016.00053

**Published:** 2016-05-10

**Authors:** Dylan J. H. A. Henssen, Erkan Kurt, Tamas Kozicz, Robert van Dongen, Ronald H. M. A. Bartels, Anne-Marie van Cappellen van Walsum

**Affiliations:** ^1^Department of Anatomy, Donders Institute for Brain Cognition and Behavior, Radboud University Medical CenterNijmegen, Netherlands; ^2^Department of Neurosurgery, Radboud University Medical CenterNijmegen, Netherlands; ^3^Department of Anaesthesiology, Pain and Palliative Care, Radboud University Medical CenterNijmegen, Netherlands

**Keywords:** trigeminal nerve, trigeminothalamic tract, orofacial pain, trigeminal neuropathy, bilateral registration

## Abstract

Orofacial pain in patients relies on the anatomical pathways that conduct nociceptive information, originating from the periphery towards the trigeminal sensory nucleus complex (TSNC) and finally, to the thalami and the somatosensorical cortical regions. The anatomy and function of the so-called trigeminothalamic tracts have been investigated before. In these animal-based studies from the previous century, the intracerebral pathways were mapped using different retro- and anterograde tracing methods. We review the literature on the trigeminothalamic tracts focusing on these animal tracer studies. Subsequently, we related the observations of these studies to clinical findings using fMRI trials. The intracerebral trigeminal pathways can be subdivided into three pathways: a ventral (contralateral) and dorsal (mainly ipsilateral) trigeminothalamic tract and the intranuclear pathway. Based on the reviewed evidence we hypothesize the co-existence of an ipsilateral nociceptive conduction tract to the cerebral cortex and we translate evidence from animal-based research to the human anatomy. Our hypothesis differs from the classical idea that orofacial pain arises only from nociceptive information via the contralateral, ventral trigeminothalamic pathway. Better understanding of the histology, anatomy and connectivity of the trigeminal fibers could contribute to the discovery of a more effective pain treatment in patients suffering from various orofacial pain syndromes.

## Introduction

Facial pain can be caused by many factors. One of the most severe and highly incapacitating conditions in which pharmacological treatments have an insufficient effect, are called trigeminal neuropathies, clinically often known as trigeminal neuralgia (Tsubokawa et al., 1991, 1993; Nguyen et al., 2000; Raslan et al., 2011; Slotty et al., 2015; Kolodziej et al., 2016). Although trigeminal neuropathies were first described more than 300 years ago, little is known about the relationship with the trigeminal nerve itself and the origin of the pain (Burchiel, 2003; Jantsch et al., 2005). In order to gain insight in the pathophysiology of trigeminal neuropathy, the anatomical connections between the trigeminal nerve and the involved brain regions seem of great importance. In summary, it is generally believed that sensory fibers involved in the conduction of pain and temperature spread over the trigeminal sensory nucleus complex (TSNC) and then cross over to the contralateral thalamus and cerebral cortex (Greenspan and Winfield, 1992; Bushnell et al., 1999; Kanda et al., 2000; Nieuwenhuys et al., 2008)****. In 2010, however, Nash et al. (2010) reported a bilateral fMRI registration in humans after noxious orofacial stimulation. Twenty-eight human subjects were injected with hypertonic saline (0.3 ml) into the central belly of the right masseter muscle and into the overlaying skin. Using blood oxygen level dependent (BOLD) contrast, a 3T Scanner imaged a bilateral fMRI-activation of the thalamus, S1 and S2 cortices after noxious orofacial stimulation. As an explanation, the authors hypothesized an extra tract, originating from the trigeminal nuclei running towards both thalami. However, no anatomical details about topography, explanation or evidence can be found in the anatomical literature for this hypothesized extra tract. The aims of this review are: (1) to provide a detailed overview of existing knowledge of the anatomy and function of the trigeminal nerve, its nuclei and its intracerebral pathways in animals; (2) to present studies that use functional imaging in the discussion of cortical representation of pain; and (3) to gain new insights in trigeminal anatomy in humans by synthesizing animal-based studies and papers that discuss functional imaging in humans.

## Anatomy of the Trigeminal Nerve and the TSNC

**Figure 1:**
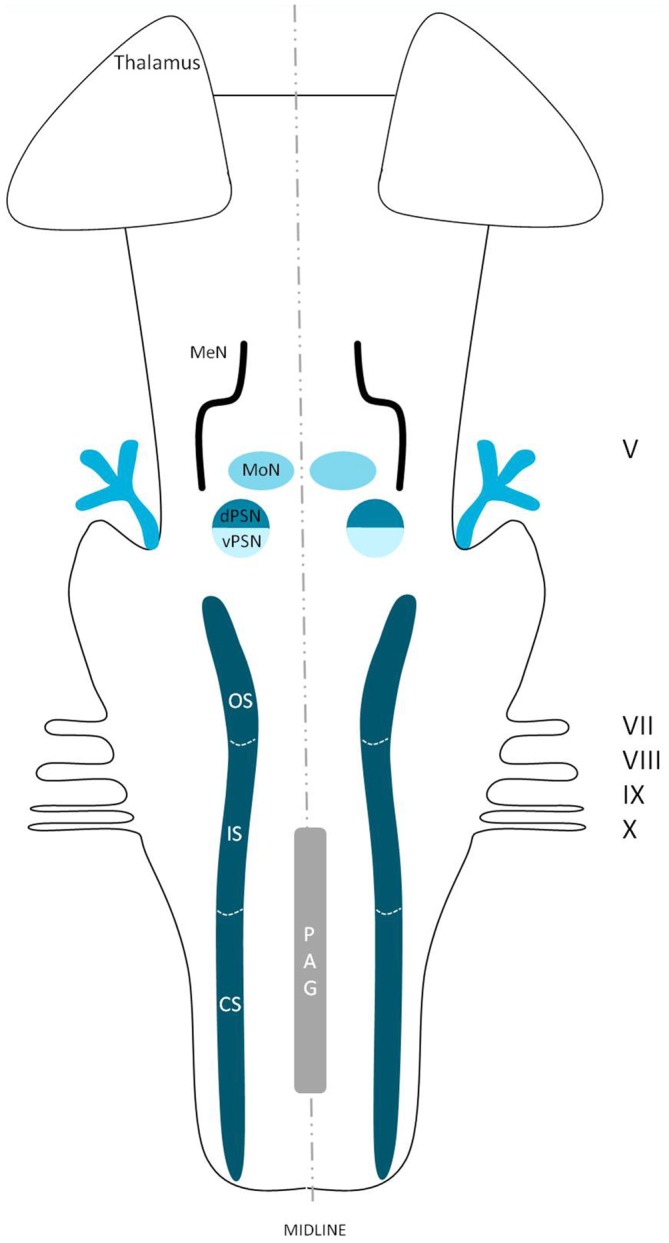
**Schematic overview of the trigeminal nuclei in the brainstem.** MeN, Mesencephalic nucleus; MoN, Motor nucleus; dPSN, Dorsal part of the principal sensory nucleus; vPSN, Ventral part of the principal sensory nucleus; OS, Oral part of the spinal nucleus; IS, Interpolar part of the spinal nucleus; CS, Caudal part of the spinal nucleus; PAG, Periaquaductal gray; which receives afferents and courses more cranially as the intranuclear tract. V, Trigeminal nerve; VII, Facial nerve; VIII, Vestibulocochlear nerve; IX, Glossopharyngeal nerve; X, Vagus nerve.

The extracerebral portion of the three divisions of the trigeminal nerve (V1: ophthalmic division, V2: maxillary division, V3: mandibular division) has been described extensively before by many authors (Lang, 1981; Usunoff et al., 1997; Sessle, 2000; Go et al., 2001; Williams et al., 2003; Schünke et al., 2006; Nieuwenhuys et al., 2008; Borges and Casselman, 2010; Sabancl et al., 2011; Bathla and Hegde, 2013; Joo et al., 2014; Marur et al., 2014). The three main divisions fuse at the trigeminal ganglion, which divides into motor and sensor rootlets. These rootlets enter the lateral pons and fibers course towards the four trigeminal nuclei: the (1) Principal Sensory Nucleus (PSN); (2) Mesencephalic Nucleus (MeN); (3) Spinal Nucleus (SN); and (4) Motor Nucleus (MoN; Figure 1). The PSN and the SN together are also called the trigeminal sensory nuclear complex (TSNC) and are held responsible for the conduction of pain and temperature information (Matsushita et al., 1982).

**Figure 2:**
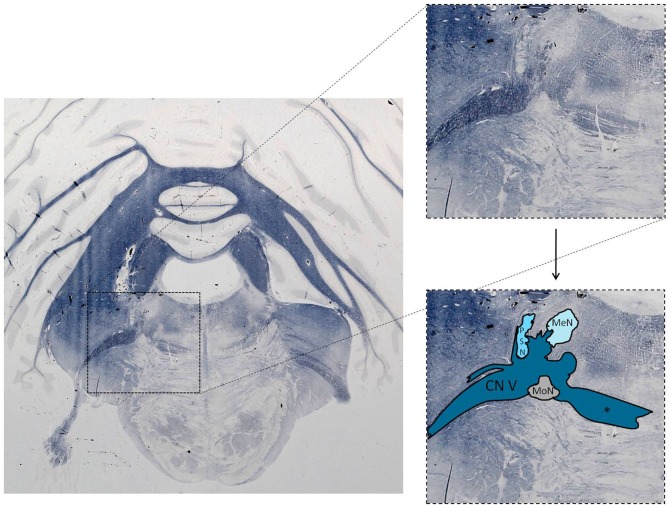
**Histological example of the anatomy of the trigeminal sensory nucleus complex (TSNC).** Histological section stained for myelin with the modified Heidenhain-Woelcke stain at different levels of the cerebellum and the pons. The unstained areas are recognized as nuclei. CN V, Trigeminal nerve; MeN, Mesencephalic nucleus; PSN, Principal sensory nucleus of the trigeminal nerve; MoN, Motor nucleus of the trigeminal nerve. *Decussating fibers of the trigeminal nucleus. The spinal nucleus is not visible is this section. Unpublished data, published with consent of Mollink et al. (2015).

**Figure 3:**
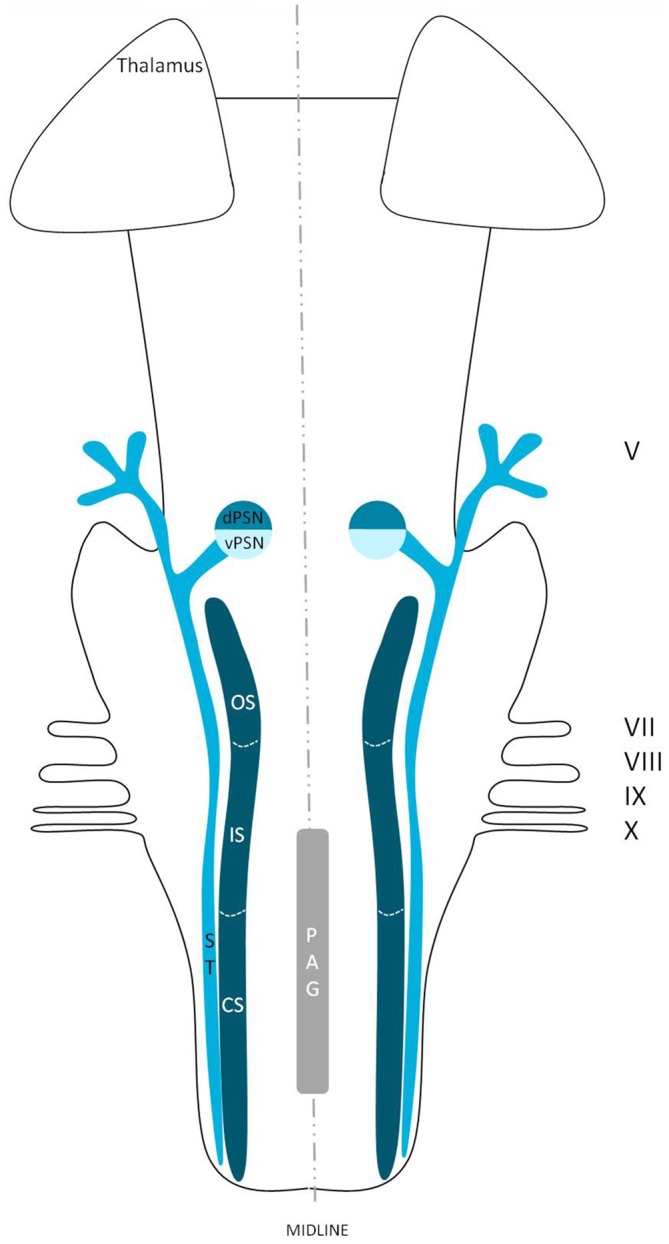
**Anatomy of the different trigeminal nuclei that are part of the TSNC.** dPSN, Dorsal part of the principal sensory nucleus; vPSN, Ventral part of the principal sensory nucleus; OS, Oral part of the spinal nucleus; IS, Interpolar part of the spinal nucleus; CS, Caudal part of the spinal nucleus; ST, Spinal tract; PAG, Periaquaductal gray; which receives afferents and courses more cranially as the intranuclear tract. V, Trigeminal nerve; VII, Facial nerve; VIII, Vestibulocochlear nerve; IX, Glossopharyngeal nerve; X, Vagus nerve.

The trigeminal nuclei have been well described by many authors (Ramon y Cajal, 1909; Meessen and Olszewski, 1949; Olszewski, 1950; Astrom, 1953; Taber, 1961; Eisenmann et al., 1963). A histological example of all the trigeminal nuclei, except the SN, is presented in Figure 2. This histological blockface was obtained from the unpublished materials from Mollink et al. (2015) and with consent of the authors adapted and published here. The literature concerning the TSNC, is summarized below. The PSN or pontine nucleus of the trigeminal nerve is located dorsolaterally to the motor nucleus of the trigeminal nerve in the pons. Its afferent fibers contribute to the perception of discriminative sensations. The termination of these afferent fibers can be divided into a ventral and a dorsal projection site within the PSN. The PSN in the cat is a compact formation and consists of different shapes of neurons (round, stellar and triangular types; Gobel and Dubner, 1969). According to Kiknadze et al. (2001) however, the subdivision of the PSN into a ventral and dorsal part is arguable. Analysis of their data shows different-sized and different-shaped neurons throughout the entire nucleus, at equal frequencies. The SN is located medially to the descending spinal tract (ST) which is located in the dorsolateral region of the brainstem. The ST extends from the trigeminal entry zone (middle pons) to the third cervical spinal cord segment. The SN therefore is oriented in a longitudinal plane and can be subdivided into three subnuclei: the caudal (CS), interpolar (IS) and oral (OS) subnucleus. The CS extends from C3 to the obex and seems consistent with the dorsal horn of the cervical spinal cord. Due to this consistency, the subdivision into lamina according to Rexed (1952, 1954) can be used. Trigeminothalamic fibers are found in the layers I, V and VI of the CS and are thought to provide the anatomical and physiological substrate for pain and temperature perception in the facial region (Dubner et al., 1978). The IS on the other hand can be found in between the CS end of the obex and the CS part of the motor nucleus of the facial nerve. The medial and rostral borders have been described to be difficult to recognize under the light microscope (Capra and Dessem, 1992). The exact function remains unclear, but it is known that the IS enlarges when the vibrissae in rodents are well developed and therefore have a heavy central representation. The IS can be subdivided into different regions, receiving input from different terminal branches of the trigeminal nerve. The dorsolateral region receives input from the auriculotemporal nerve, whereas the ventrolateral region is the termination zone of the other ophthalmic and maxillary branches (Jacquin et al., 1983; Capra and Dessem, 1992). The OS or rostral subnucleus extends from the CS pole of the facial motor nucleus to the CS part of the motor nucleus of the trigeminal nerve (Crosby and Yoss, 1954). This subnucleus can be subdivided into a lateral and medial part. The medial subdivision receives afferents from intraoral structures, whereas the lateral part of the OS registers information from the dorsal structures of the face and the vibrissae (Eisenmann et al., 1963). Figure 3 recapitulates the anatomy of the different trigeminal nuclei that are part of the TSNC.

## Intracerebral Anatomy of Fibers Originating from the TSNC

### Efferents from the PSN

**Figure 4:**
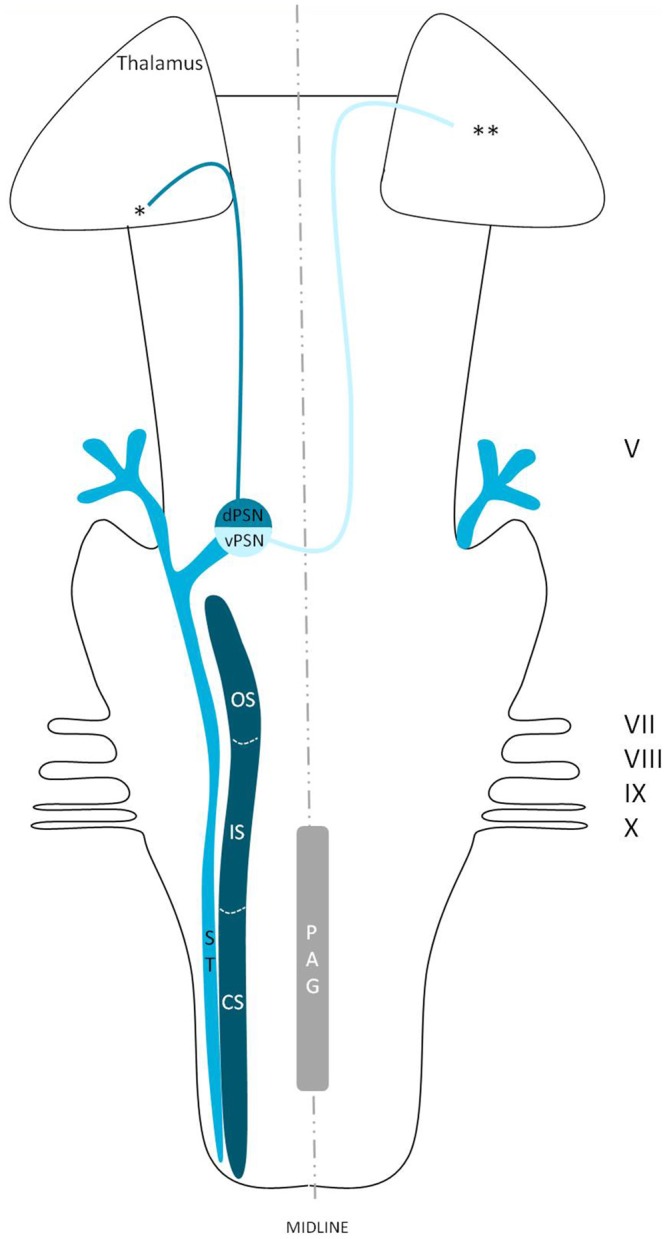
**Anatomy of the trigeminothalamic tracts sprouting from the principal sensory nucleus within the brainstem towards the diencephalon.** dPSN, Dorsal part of the principal sensory nucleus; vPSN, Ventral part of the principal sensory nucleus; OS, Oral part of the spinal nucleus; IS, Interpolar part of the spinal nucleus; CS, Caudal part of the spinal nucleus; ST, Spinal tract; PAG, Periaquaductal gray; which receives afferents and courses more cranially as the intranuclear tract. V, Trigeminal nerve; VII, Facial nerve; VIII, Vestibulocochlear nerve; IX, Glossopharyngeal nerve; X, Vagus nerve. *Dorsal trigeminothalamic tract; **Ventral trigeminothalamic tract.

Wallenberg (1905) dissected the brains of rabbits and observed uncrossed trigeminothalamic fibers, sprouting from the dorsal part of the PSN. After the example of Wallenberg (1905) others mentioned this ipsilateral circuit as well (Economo, 1911; Woodburne, 1936; Papez and Rundles, 1937; Walker, 1939; Papez, 1951; Carpenter, 1957) . Torvik (1957) studied the ascending pathways of the trigeminal nerve by means of a partial or complete transection of the rostral brains of 22 kittens and retrograde cellular alterations in the TSNC. It was concluded that from the PSN almost all fibers projected to one of both thalami and that these projections were both contralateral as ipsilateral. Smith (1975) carried out a partial unilateral stereotactic lesion of the PSN in cebus and rhesus monkeys and found a ventromedial decussation of fibers at the level of the pontine tegmentum and a dorsal collection of axons that form a smaller trigeminothalamic projection, originating from the dorsal one-third of the PSN. No neurons from the PSN appeared to project to the spinal cord (Matsushita et al., 1982). Matsushita et al. (1982) also used the retrograde horseradish peroxidase technique and injected it into the posterior ventral nucleus of the thalamus. A large number of neurons were observed in the ventral segment of the PSN and the IS of the SN on the contralateral side, whereas on the ipsilateral side, the dorsal aspect of the PSN was marked after injection. Rausell and Jones (1991) bilateral afferents to the VPM, originating from both the ipsilateral and the contralateral PSN using an anterograde tracing study in 3 cynomolgous monkeys (*Macaca fascicularis*). Table 1 summarizes the mentioned tracing studies in animals. Figure 4 depicts the trigeminothalamic tracts sprouting from the PSN.

**Table 1:** **Tracing studies of the principal sensory nucleus (PSN)**.

Reference	Species	Tracing technique	Anatomical site of lesion/injection
Wallenberg (1905)	Rabbit	Marchi method after lesion	PSN
Economo (1911)	Macaque monkey	Degeneration	PSN
Woodburne (1936)	Series of vertebrates	Chroom silver preparation after sectioning	Section staining
Walker (1939)	Rhesus monkey	Marchi method	PSN
Papez (1951)	Series of quadrupeds	Weigert-Pal method	Section staining
Carpenter (1957)	Rhesus monkey	Marchi method after lesion	SCP/Mesencephalon
Torvik (1957)	Cat	Degeneration after lesion	PSN
Smith (1975)	Cebus monkey	Variety of Nauta silver	PSN
	Rhesus monkey	impregnations	
Matsushita et al. (1982)	Cat	Horseradish peroxidase	Posterior ventral nucleus of the thalamus
Rausell and Jones (1991)	Cynomolgus monkey	Horseradish peroxidase;	Anterograde: CS
		Germ agglutin-conjugated horseradish peroxidase;	Retrograde: S1-cortex, facial area
		Solution of 5% fast blue

*PSN, Principal sensory nucleus; SCP, Superior cerebellar peduncle*.

### Efferents from the SN

**Figure 5:**
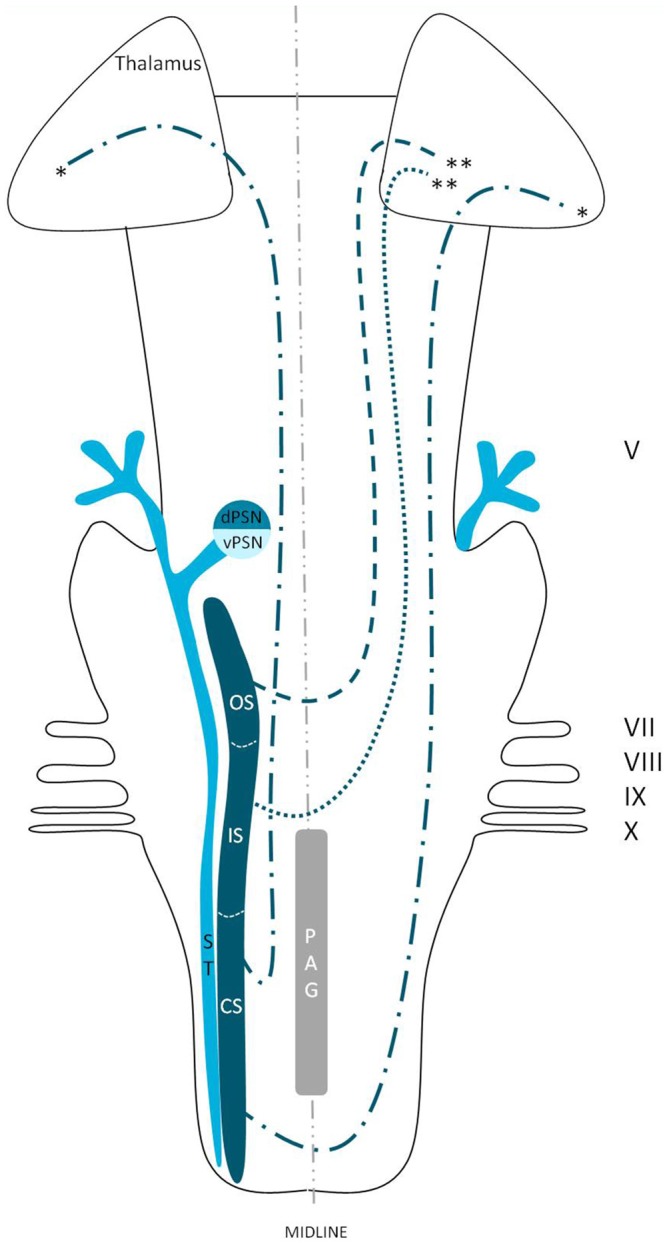
**Anatomy of the trigeminothalamic tracts sprouting from the spinal nucleus within the brainstem towards the diencephalon.** dPSN, Dorsal part of the principal sensory nucleus; vPSN, Ventral part of the principal sensory nucleus; OS, Oral part of the spinal nucleus; IS, Interpolar part of the spinal nucleus; CS, Caudal part of the spinal nucleus; ST, Spinal tract; PAG, Periaquaductal gray; which receives afferents and courses more cranially as the intranuclear tract. V, Trigeminal nerve; VII, Facial nerve; VIII, Vestibulocochlear nerve; IX, Glossopharyngeal nerve; X, Vagus nerve. *Dorsal trigeminothalamic tract; **Ventral trigeminothalamic tract.

**Figure 6:**
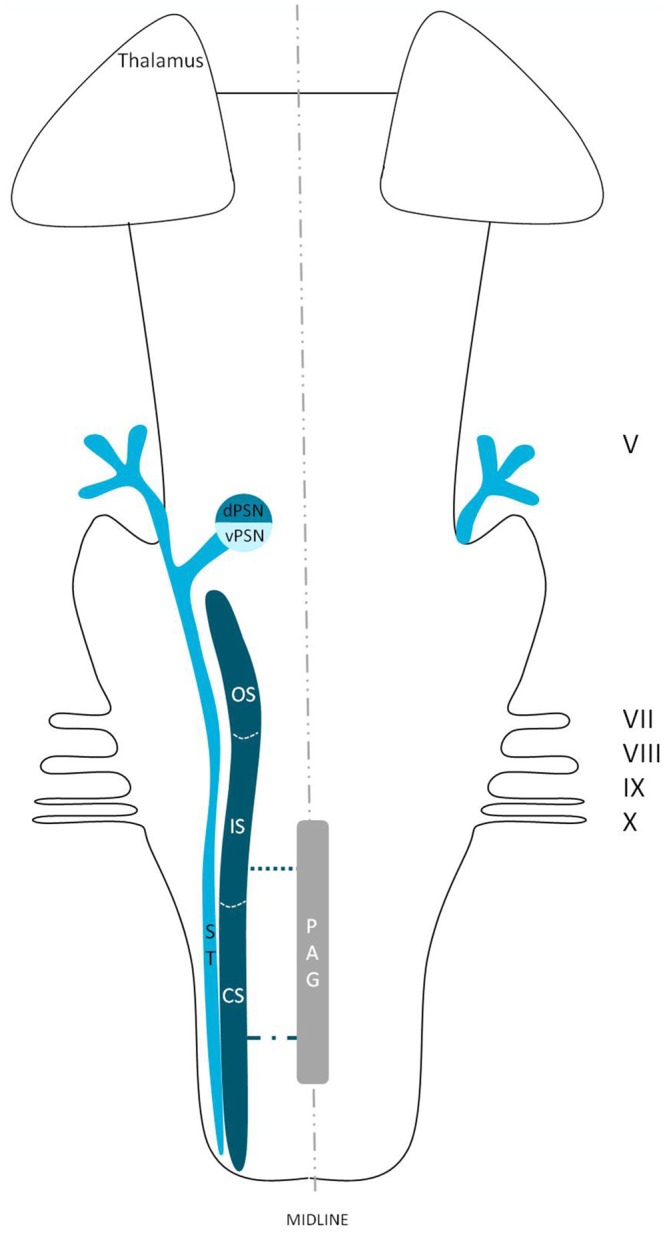
**Anatomy of the intranuclear tract within the brainstem.** dPSN, Dorsal part of the principal sensory nucleus; vPSN, Ventral part of the principal sensory nucleus; OS, Oral part of the spinal nucleus, IS, Interpolar part of the spinal nucleus; CS, Caudal part of the spinal nucleus; ST, Spinal tract; PAG, Periaquaductal gray; which receives afferents and courses more cranially as the intranuclear tract. V, Trigeminal nerve; VII, Facial nerve; VIII, Vestibulocochlear nerve; IX, Glossopharyngeal nerve; X, Vagus nerve.

Ganchrow (1978) injected the CS of the SN with tritiated amino acids in the squirrel monkey and found that the efferents from the CS had a contralateral projection to the VPM. Also, bilateral connections were observed to the mediodorsal nucleus (MD), together with ipsilateral connections between the PSN and the CS of the SN. Burton et al. (1979) studied the projections from the CS of the spinal trigeminal complex with retrograde and anterograde axonal transport techniques in cats. Projections to the thalamus were both bilaterally to a dorsomedial region of the VPM as well as contralaterally to the main part of the VPM and PO (posterior nucleus) of the thalamus. Künzle (1998) a weak bilateral projection from the CS of the SN in the hedgehog tenrec (Echinops telfairi) after injection of trigeminal subdivisions with wheat germ agglutinin conjugated to horseradish peroxidase, biotinylated dextran amine and a solution of radioactive amino acids. There was little evidence for a trigeminal projection to the intralaminar nuclei but there was a distinct projection to the contralateral zona incerta of the thalamus. Furthermore, Ikeda et al. (1982) described intranuclear ascending fibers originating from the IS of the cat, after applying injections into the SN. The OS of the SN has been described to be consistent with the PSN. Efferents originating from the OS of the SN cross over to the contralateral VPM as a part of the trigeminal lemniscus (Nieuwenhuys et al., 2008). This would result in the trigeminothalamic tract sprouting from the SN as depicted in Figure 5. Furthermore, Panneton and Burton (1982) injected retrograde horseradish peroxidase into the rostral trigeminal region and showed that neurons in all laminae, however mainly III and IV of the medullary dorsal horn, project through an intranuclear pathway. Within layer III and IV orofacial fibers converge into their separate nuclei. Also, layer III and IV contain, next to orofacial fibers and trigeminal nuclei, many interneurons that can be responsible for the intranuclear pathway (Dubner et al., 1978). A third tract therefore can be described, the so-called intranuclear tract running towards or within the PAG from the IS and CS of the SN. This would result in the trigeminothalamic tract as depicted in Figure 6.

Table 2 provides an overview of the mentioned tracing studies.

**Table 2:** **Tracing studies of the spinal nucleus (SN)**.

Reference	Species	Tracing technique	Anatomical site of lesion/injection
Ganchrow (1978)	Squirrel monkey	Degeneration after lesion; Injection with tritiated amino acids	CS
Burton and Craig (1979)	Cat and Cynomolgus	Injection with horseradish peroxidase	Ventroposterior nucleus of the thalamus
	monkey		
Burton et al. (1979)	Cat	Injection with mixture of amino acids; Injection with horseradish peroxidase	Anterograde: CS Retrograde: ventroposterior nucleus of the thalamus
Ikeda et al. (1982)	Cat	Injection with horseradish peroxidase	IS
Panneton and Burton (1982)	Cat	Injection with horseradish peroxidase	Rostral trigeminal region
Künzle (1998)	Hedgehog tenre	Injection with a mixture of wheat germ agglutinin conjugated to horseradish peroxidase, biotinylated dextran amine and a solution of radioactive aminoacids	SN and PSN
Negredo et al. (2009)	Sprague-Dawley rat	Injection with dextran amine	Thalamus

*CS, Caudal subnucleus; IS, Interpolar subnucleus; SN, Spinal nucleus; PSN, Principal sensory nucleus*.

## Function of the Dorsal Trigeminothalamic Tract

**Figure 7:**
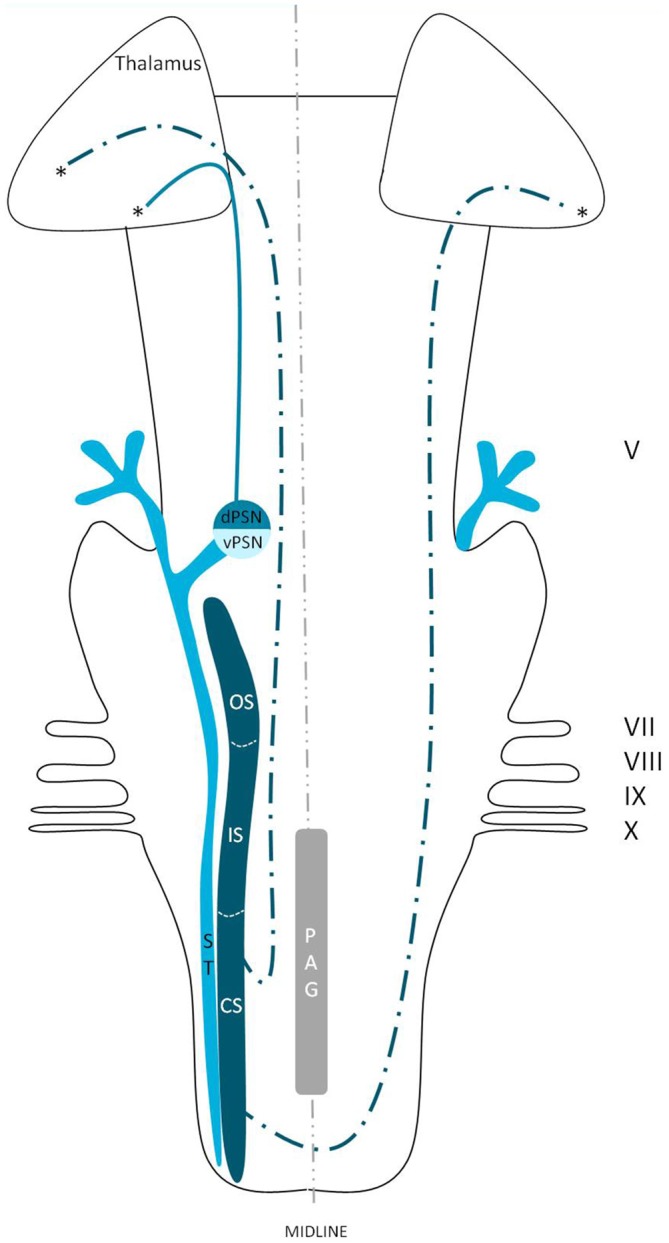
**Anatomy of the dorsal trigeminothalamic tract within the brainstem towards the diencephalon.** dPSN, Dorsal part of the principal sensory nucleus; vPSN, Ventral part of the principal sensory nucleus; OS, Oral part of the spinal nucleus; IS, Interpolar part of the spinal nucleus; CS, Caudal part of the spinal nucleus; ST, Spinal tract; PAG, Periaquaductal gray; which receives afferents and courses more cranially as the intranuclear tract. V, Trigeminal nerve; VII, Facial nerve; VIII, Vestibulocochlear nerve; IX, Glossopharyngeal nerve; X, Vagus nerve. *Dorsal trigeminothalamic tract.

Although the trigeminothalamic connections and origins have extensively been described, little is known about the cells giving rise to these tracts. The dorsal trigeminothalamic tract in animals (cats and monkeys) consists of fibers originating from the dorsal PSN and the CS and OS of the SN (Burton and Craig, 1979; Matsushita et al., 1982; Nieuwenhuys et al., 2008). This is summarized in Figure 7. The dorsal PSN receives afferents originating from the oral cavity, hence it is associated with the intraoral sensitivity (Shigenaga et al., 1986). Takemura et al. (1993) by studying the afferent axons from the lower and upper teeth. They found that these fibers project to the PSN in monkeys. According to some authors, the PSN also receives mechanoreceptive afferents from the intraoral cavity (Zeigler and Witkovsky, 1968; Silver and Witkovsky, 1973; Kishida et al., 1985; Dubbeldam, 1998). In line with these studies, bird species that rely on tactile information while feeding, the complete PSN seems to be enlarged (Gutiérrez-Ibáñez et al., 2009). Shigenaga et al. (1986) showed that in cats, the branches supplying the anterior face, i.e., the frontal, infraorbital and mental nerves, also terminate in the ventral PSN. Furthermore, the alveolar (superior and inferior), buccal, lingual and pterygopalatine branches, responsible for the intraoral sensitivity, terminate not only in different areas of the PSN but also in the OS and IS of the SN. The IS of the SN also receives input from the anterior face region and the auriculotemporal, corneal, mylohyoid, and zygomatic afferent nerve fibers (Shigenaga et al., 1986). The projecting cells from the CS of the SN are held responsible for the transmission of pain and temperature from the orofacial region. However, dental pulp afferents projecting to the OS of the SN have also been described (Burton and Craig, 1979; Takemura et al., 1993). The afferents of the OS of the SN are described to convey noxious information after mechanical stimulation (Woda et al., 1977), but the OS has also been described as a CS extension of PSN (Eisenmann et al., 1963; Burton et al., 1979). Others described that the terminals from both the upper and lower pulpal afferents formed a connection between the PSN and the OS of the SN (Takemura et al., 1993). The IS and CS also receive afferents from the intra-oral cavity, though this projection is less dense compared to that of the PSN and the OS of the SN (Takemura et al., 1993). Therefore, the exact function of these separate subnuclei remains unclear. However, most assume that the ipsilateral, dorsal trigeminothalamic tract is responsible for proprioceptic sensorical information, it seems logical to assume that both the SN and the PSN receive pain, temperature and mechanoreceptive stimuli from the head and intraoral cavity.

## Function of the Contralateral, Ventral Trigeminothalamic Tract

**Figure 8:**
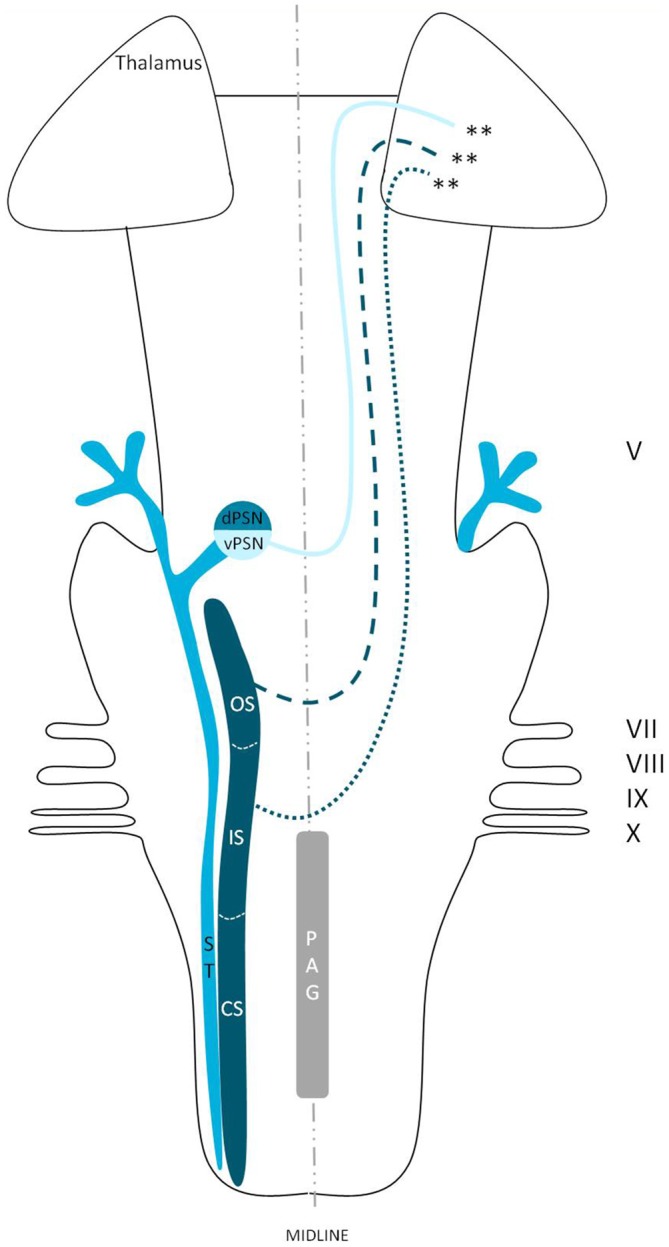
**Anatomy of the ventral trigeminothalamic tract within the brainstem towards the diencephalon.** dPSN, Dorsal part of the principal sensory nucleus; vPSN, Ventral part of the principal sensory nucleus; OS, Oral part of the spinal nucleus; IS, Interpolar part of the spinal nucleus; CS, Caudal part of the spinal nucleus; ST, Spinal tract; PAG, Periaquaductal gray; which receives afferents and courses more cranially as the intranuclear tract. V, Trigeminal nerve; VII, Facial nerve; VIII, Vestibulocochlear nerve; IX, Glossopharyngeal nerve; X, Vagus nerve. **Ventral trigeminothalamic tract.

The ventral trigeminothalamic tract, as depicted in Figure 8, consists of fibers originating from the ventral PSN, CS and IS of the SN. The fibers from this ventral tract decussate along the medial border of the medial lemniscus and are therefore also called the trigeminal lemniscus (Torvik, 1957; Smith, 1975; Matsushita et al., 1982; Nieuwenhuys et al., 2008). The ventral trigeminothalamic tract is held responsible for the conduction of vital information. The function of the various nuclei has been studied intensively before. The PSN is believed to be mainly involved in the conduction of tactile sensations and movement or position sense (Kruger, 1979). However, Kiknadze et al. (2001) showed that the same nucleus is also involved in the processing of orofacial and dental pain in cats. According to Shigenaga et al. (1986) the IS of the SN also receives input from the anterior orofacial region and several trigeminal peripheral branches. As we know from Sjögvist’s tractotomy, the CS plays an important role in the transmission of vital information (Sjöqvist, 1938). These results would suggest that the ventral trigeminothalamic tract plays an important role in the contralateral registration of orofacial nociception, as suggested before by others (Sessle, 2000; Nieuwenhuys et al., 2008).

## Activation of Brain Regions in Response to Orofacial Noxious Stimulation

Pain, including that of orofacial origin, can be mediated by two systems. The medial system is composed of limbic structures and the anterior cingulate and insular cortices and is responsible for the emotional-affective and cognitive-behavioral dimensions of pain (Kulkarni et al., 2005; Wiech et al., 2006). The lateral pain network consists of the lateral spinothalamic tract, the VPL or VPM of the thalamus and the S1 cortex and processes the sensory-discriminative components of pain (Kenshalo et al., 1988; Bushnell and Duncan, 1989; Bushnell et al., 1999). The main components of the acute pain network are the prefrontal, M1, S2, anterior cingulate and insular cortices, the thalamus, supplementary motor areas, amygdala, PAG and basal ganglia (Apkarian et al., 2005). According to classical knowledge, it would be logical to assume that contralateral activation of the lateral system in response to unilateral noxious stimulation would occur. Surprisingly, according to Peyron et al. (2000) bilateral hemodynamic responses to acute noxious stimuli were observed in the thalamus and anterior cingulate, insular and SII cortices. An activation of S1, prefrontal and posterior parietal cortices, the striatum, cerebellum, PAG and supplementary motor areas was observed contralateral to the stimulus (Peyron et al., 2000). Bingel et al. (2004a,b) published a bilateral somatotopic cortical registration in event related fMRI after painful stimulation of the hand and foot. Touchless laser pain stimuli were applied to the dorsum of the hand and foot after which the neuronal response was measured using BOLD fMRI. In general, Bingel et al. (2004a,b) concluded that ipsilateral activity of S1 could be the result of an uncrossed ipsilateral tract or transcallosal excitatory pathways. Farrell et al. (2005) reviewed the literature on upper extremity noxious stimulation and showed a predominant contralateral activation of the anterior cingulate cortex (ACC), lentiform nucleus and the S1, S2 and M1 cortices, however the included reports discussed various activation patterns of cortical and subcortical structures. An ispilateral activation of the midbrain was also observed. The insular cortex, thalamus, cerebellum, premotor areas and inferior parietal lobule were regions that showed bilateral activation after noxious stimulation of the upper extremity.

Taking orofacial pain into account, in May et al. (1998) injected capsaicin in the foreheads of seven healthy volunteers. May et al. (1998) showed a bilateral activation of the cerebellum and the anterior insula and observed ipsilateral activation of the ACC and contralateral activation of the thalamus. DaSilva et al. (2002) showed an ipsilateral activation of the SN in patients that underwent noxious thermal stimulation of the skin of the trigeminal areas (V1, V2 and V3). Also, DaSilva et al. (2002) showed a contralateral activation of the thalamus and S1 cortex after stimulation. Brooks et al. (2005) showed a bilateral activation of the anterior insula, S2 and a contralateral activation of the posterior insula after noxious thermal stimulation of the face, hand or foot. When stimulating the face or hand, thalamic activity was also observed. Jantsch et al. (2005) discussed a bilateral fMRI activation of the S1 cortices after painful dental stimulation in eight healthy subjects. Interestingly, Jantsch et al. (2005) also mention a significant increase of BOLD-activation in the ipsilateral hemisphere after stimulation for which they do not give any explanation. Jantsch et al. (2005) that a complex cortical network must be responsible for a bilateral activation after orofacial stimulation. de Leeuw et al. (2006) observed brain activation with painful hot stimulation of the trigeminal nerve. In nine participants, the skin overlaying the left masseter muscle was triggered using thermal stimuli. Using fMRI, brain activity was registered. Bilateral activation was seen in the ACC, insula and thalamus. Ettlin et al. (2009) reported that bilateral non-nociceptive orofacial mechanical stimulation can provoke a bilateral activation of the insular cortex, whereas the S1-cortex was rarely activated. In the same year, Nash et al. (2009) investigated nociception in 30 humans (22 males, 19–52 years) using painful saline injections in the right masseter muscle. Both cutaneous and muscle nociceptive input activated the CS and OS subdivision of the SN. However, cutaneous nociceptive stimulation evoked a large response within the IS part of the SN, whereas muscle nociception was registered in the PSN. Weigelt et al. (2010) studied thirteen healthy volunteers that underwent stimulation of the dental pulp with a constant current tooth stimulator. After stimulation, they reported a bilateral activation of the S1, S2, the medial dorsal nuclei of the thalamus, insular cortices, ACC and precentral areas such as M1 as seen on fMRI. The information of the studies that discuss orofacial pain is presented in Table 3.

**Table 3:** **Synopsis of activated brain areas after noxious stimulation**.

Reference	Site of stimulation	Medial dorsal thalamus	S1	S2	ACC	Insular cortical regions	Precentral gyrus
May et al. (1998)	Subcutaneous capsaicin injection into the forehead	Contralateral	N/A	N/A	Ipsilateral	Bilateral^B^	N/A
DaSilva et al. (2002)	Cutaneous thermal stimulation of right V1 region	Contralateral	Contralateral	N/A	N/A	N/A	N/A
	Cutaneous thermal stimulation of right V2 region	Contralateral	Contralateral	N/A	N/A	N/A	N/A
	Cutaneous thermal stimulation of right V3 region	Contralateral	Contralateral	N/A	N/A	N/A	N/A
Brooks et al. (2005)	Cutaneous thermal stimulation of the area below the right lower lip	Small activations^E^	N/A	Bilateral	N/A	Contralateral^D^	N/A
	Cutaneous thermal stimulation of the dorsum of the right hand	Small activations^E^	N/A	Bilateral	N/A	Contralateral^D^	N/A
	Cutaneous thermal stimulation of the dorsum of the right foot	N/A	N/A	Bilateral	N/A	Contralateral^D^	N/A
Jantsch et al. (2005)	Pneumatic mechanical stimulation of the middle phalanx	N/A	Contralateral^A^	Bilateral	Bilateral	Bilateral	Contra-lateral
	Constant electrical dental stimulation	N/A	Bilateral	Bilateral	Bilateral	Bilateral	Contra-lateral
de Leeuw et al. (2006)	Cutaneous thermal stimulation of the skin area overlying the left masseter muscle	Bilateral	Contra-lateral	N/A	Bilateral	Bilateral	Ipsilateral
Ettlin et al. (2009)	Electrical dental stimulation of one randomly selected canine with randomized intervals	N/A	N/A	N/A	Small activations^E^	Small activations^B,E^	Small activations^E^
Nash et al. (2010)	Subcutaneous hypertonic saline injection into the skin overlying the right masseter muscle and into the central belly of the right masseter muscle	Bilateral	Bilateral	Bilateral	N/A	N/A	N/A
Weigelt et al. (2010)	Constant electrical pulpal stimulation	Bilateral	Bilateral	Bilateral	Bilateral	Bilateral	Bilateral

*N/A, not available from full text; ^A^Hand-area on S1; ^B^Anterior insular cortex; ^C^Medial insular cortex; ^D^Posterior insular cortex; ^E^Author does not specify the results*.

When investigated, the S2, insular and cingulate cortices seemed to be part of a bilateral projection system. Other structures, such as the thalamus, S1 cortex and the precentral gyrus, were also involved in the bilateral pain registration (Jantsch et al., 2005; de Leeuw et al., 2006; Staud et al., 2007; Cole et al., 2010; Weigelt et al., 2010). Nevertheless, Brügger et al. (2011) subdivided three lateralization patterns in the brain related to processing dental pain: (1) hemispheric lateralization irrespective of side of stimulation; (2) structures with predominant contralateral activation; and (3) structures showing hemispheric dominance and predominant contralateral activation. Pattern 1 shows that the right hemispheric effect is stronger to the cerebellar lobes and the parahippocampal area. The left hemispheric effect on the other hand is stronger to the putamen, pregenual, posterior and anterior cingulate cortices and supramarginal area. The second pattern shows five brain areas that are predominantly contralateral: the S1-cortex, thalamus, posterior insula, amygdala, and subcentral area. The subcentral area also shows lateralization to one hemisphere according to pattern 3. Also, they observe an activation of the contralateral amygdala in response to noxious dental stimulation.

## Discussion

**Figure 9:**
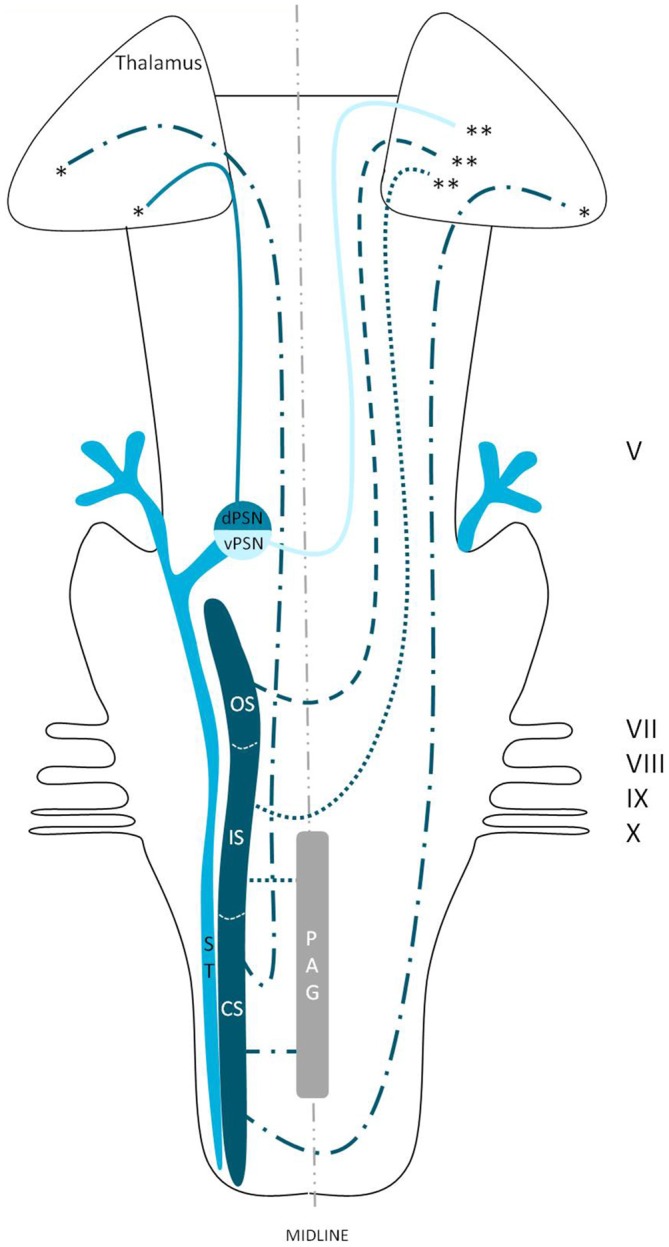
**Anatomy of the hypothesized bilateral orofacial pain registration system in humans.** dPSN, Dorsal part of the principal sensory nucleus; vPSN, Ventral part of the principal sensory nucleus; OS, Oral part of the spinal nucleus; IS, Interpolar part of the spinal nucleus; CS, Caudal part of the spinal nucleus; ST, Spinal tract; PAG, Periaquaductal gray; which receives afferents and courses more cranially as the intranuclear tract. V, Trigeminal nerve; VII, Facial nerve; VIII, Vestibulocochlear nerve; IX, Glossopharyngeal nerve; X, Vagus nerve. *Dorsal trigeminothalamic tract; **Ventral trigeminothalamic tract.

We reviewed in animals that the somatosensory fibers of the fifth cranial nerve are distributed over the TSNC. From these nuclei, three tracts can be recognized. From the ventral part of the PSN, a large crossed tract, the trigeminal lemniscus or the ventral trigeminothalamic tract arises. This tract also receives efferents originating from the OS and IS of the SN. From the dorsal part of the PSN arises the dorsal trigeminothalamic tract, which also consists out of fibers from both the contra- and ipsilateral SN. Both tracts run to the thalamus, the VPL-region in specific. A third tract can be observed, originating from the distal two thirds of the SN. Fibers of this intranuclear tract course into the PAG (Wallenberg, 1905; Kohnstamm, 1910; Economo, 1911; Woodburne, 1936; Papez and Rundles, 1937; Walker, 1939; Papez, 1951; Torvik, 1957; Carpenter, 1957; Smith, 1975; Dubner et al., 1978; Ganchrow, 1978; Burton et al., 1979; Ikeda et al., 1982; Matsushita et al., 1982; Panneton and Burton, 1982; Rausell and Jones, 1991; Nieuwenhuys et al., 2008; Negredo et al., 2009; Figure 9). Although the mentioned ipsilateral tract has been described before, it has never been hypothesized to play a prominent role in the conduction of noxious stimulation. A full understanding of brain activation in response to nociceptive information is limited by the complexity of the multidimensional character of pain and the pain experience. Lateralization of the cortical areas involved in the medial pain system that seem predominantly active and are not influenced by the side of stimulation are the different parts of the cingulate gyrus (Brügger et al., 2011). This predominant activation could explain why fMRI studies show in some cases an ipsilateral activation. When the left cingulate gyrus gets activated after subjects are stimulated on the left side of the body, this may appear to be an ipsilateral activation pattern. Nevertheless, bilateral activation of the cingulate gyri has also been observed after unilateral noxious stimulation (Jantsch et al., 2005). The robust contralateral activation of the amygdala can only be speculated about. A high emotional value attributed to orofacial/dental pain could be one of the factors involved, but the emotional aspect of this kind of pain or noxious stimuli has never been investigated (Brügger et al., 2011). Even so, lateralization of the amygdala turns out to be inconsistent throughout human literature (Bingel et al., 2002; Bornhövd et al., 2002; Brügger et al., 2011). The subdivisions of the insular cortices showed a subdivision in activation. When bilateral activation was reported, this concerned mainly the anterior insular cortex (May et al., 1998; Jantsch et al., 2005). Contralateral activation was mainly seen in the posterior insular cortex (Brooks et al., 2005; Jantsch et al., 2005; Brügger et al., 2011). The posterior insular cortex is preferentially connected to other lateral structures, such as the S1 and S2 cortices (Wiech et al., 2014). The other structures of the lateral pain system are also predominantly contralateral according to Brooks et al.’s (2005) and Brügger et al.’s (2011) studies but this is contradicted by various reports discussing a bilateral activation (de Leeuw et al., 2006; Nash et al., 2010; Weigelt et al., 2010). The activation of the ipsilateral S1 cortex is also held implausible, according to Brügger et al. (2011), but other reports do state a bilateral activation of S1 in response to noxious stimulation of the orofacial region (Bingel et al., 2004b; Jantsch et al., 2005; Nash et al., 2010; Weigelt et al., 2010; Brügger et al., 2011). The findings (Bingel et al., 2004b; Jantsch et al., 2005; Nash et al., 2010) could be in agreement with the results from animal-based studies about the intracerebral pathways. When we focus on facial pain, a double trigeminothalamic tract could be the answer to this clinical question, if both the ventral and dorsal trigeminothalamic tract are capable of nociceptive conduction. Another anatomical solution can be found in the transcallosal pathways. Nevertheless, this seems implausible bearing in mind the study of Stein et al. (1989) in which they investigated the pain perception of a split-brain patient after high intensity noxious stimulation was applied to the foot.

Limitations in the functional imaging of pain are: (1) anticipation of pain; (2) attentional modulation; and (3) emotional accounts of pain. The anticipation of pain is known to activate several brain regions, including the ACC, cerebellum, ventral premotor and ventromedial prefrontal cortex, the PAG and hippocampus (Hsieh et al., 1999; Ploghaus et al., 1999, 2001, 2003; Bantick et al., 2002). Brügger et al. (2011) study does indeed show that, when anticipation is ruled out, the bilateral activation decreases. This decrease in bilateral activity shows that anticipation of pain causes a bilateral network to be activated. Nevertheless, when anticipation is ruled out, there still seems to be a bilateral activation of cortical areas involved in the lateral pain system. This would suggest that both systems (pain and anticipation of pain) play a prominent role in registration of pain. Secondly, it is well known that pain related anxiety and fear are associated with difficulties in attention and result in an increased awareness of pain (Taylor et al., 2015). Chronic lower back pain patients have been shown to display activation of the insular cortex, supplementary motor area and pre-motor area, cerebellum, thalamus, pulvinar, posterior cingulate cortex, hippocampus, fusiform gyrus and angular gyrus after they saw a picture showing an aversive movement (Shimo et al., 2011). The emotional accounts have been studied extensively as well. When cued expectation of pain stimuli is studied, activation of various regions within the salience (insula and ACC), sensorimotor and attentional control (parietal and frontal) networks have been described (Yágüez et al., 2005; Carlsson et al., 2006; Seidel et al., 2015). Taking the mentioned regions into account, fMRI studies of the brain can be very useful and illustrative, but one must be careful when interpreting these results.

Lin (2014) states that a critical step in the future of fMRI investigations is to understand the chronic dental pain-related anatomy and cortical representations. The potential for investigating and understanding chronic orofacial pain is highlighted by their two major findings. First, the thalamus and S1 cortex were identified as two major sites of neuroplasticity and second, the increased connectivity between the thalamus and the insula. Although some other authors also state that the standard anatomy can change under the influence of chronic stimulation, such as pain (Wilcox et al., 2013, 2015), it seems logical to assume that orofacial pain is bilaterally registered in healthy humans as well, according to other investigations (Bingel et al., 2004b; Jantsch et al., 2005; de Leeuw et al., 2006; Staud et al., 2007; Cole et al., 2010; Nash et al., 2010; Weigelt et al., 2010). In order to gain more insight in the normal connectivity from the orofacial region and the related cortical areas, we subsequently make some proposals for future investigations. A post-mortem diffusion tensor imaging (DTI) study based on a diffusion weighted MRI (DW-MRI) scan could contribute to our insights in the trigeminal fibers, because this is currently the only capable method of mapping the detailed architecture of white matter fibers in human brain specimens (Jones et al., 2013). This technique could create a more profound insight in trigeminal anatomy, specifically concerning its intracerebral portion and is certain to contribute to clinical knowledge and decision making in the daily practice of trigeminal neuropathies. Nevertheless issues regarding high resolution MRI and reliable qualitative probabilistic tracking of the trigeminothalamic tracts may be important challenges to overcome (Jones et al., 2013). Beyond the challenges inherent in acquiring suitable DW-MRI data, there are currently many obstacles to overcome regarding the tractographic modeling of white matter tracts (O’Donnell and Pasternak, 2015). The use of post-mortem DTI could be a welcome supplement to the knowledge obtained by *in vivo* fMRI-studies, in which the activation of regions of the brain involved in the orofacial pain registration, have been mapped. There still remain outstanding questions that cannot be answered today. Is it possible that the trigeminothalamic tracts in humans are more comparable to those in animals? Is it possible that the several nuclei of the TSNC are indeed part of a conjoined complex which makes it difficult to separate several types of somatosensorical information and their conducting pathways? There is much left that we do not comprehend concerning orofacial pain, but knowledge of the involved trigeminothalamic and intranuclear pathways is believed to be of great importance in treating patients suffering from orofacial pain syndromes effectively .

## Conclusion

The main aim of this review was to present new insights in trigeminal anatomy in humans, based on both animal-based papers and fMRI research studies. The classical point of view is that orofacial pain is conducted in a contralateral fashion. However by synthesizing animal-based literature and human functional imaging studies, we state that the exact neuroanatomy of orofacial pain is largely elusive, and we hypothesize the existence of a bilateral orofacial conduction system of nociceptive information in humans.

## Ethical Statement

This study was carried out in accordance with the recommendations of the CMO (Commissie Mensgebonden Onderzoek) region Arnhem-Nijmegen, Netherlands. Also, the protocol was approved by the CMO region Arnhem-Nijmegen, Netherlands. The histological blockface in Figure 2 was obtained from Mollink et al. (2015). This unpublished histological slice was acquired via the body donor program at the department of anatomy of the Radboud University Medical Centre, Nijmegen, Netherlands. All body donors in this program signed a written informed consent during lifetime permitting the use of their body and parts for science and teaching.

## Author Contributions

DJHAH undertook the action of collecting the literature and wrote the first draft of the article. After collecting multiple times the input from A-MvCvW, EK and RvD, he wrote the other versions. Together with EK, DJHAH created the Figures 2–4. Figure 1 was created by A-MvCvW and DJHAH. RHMAB and TK reviewed the latest versions and gave valuable input from their point of expertise.

## Conflict of Interest Statement

The authors declare that the research was conducted in the absence of any commercial or financial relationships that could be construed as a potential conflict of interest. Despite hosting a research topic together, the reviewer MH and handling Editor state that the process met the standards of a fair and objective review.

## Abbreviations

ACC: Anterior cingulate cortex
BOLD: Blood oxygen level dependent
CS: Caudal subnucleus
DTI: Diffusion tensor imaging
DW-MRI: Diffusion weighted magnetic resonance imaging
fMRI: Functional magnetic resonance imaging
IS: Interpolar subnucleus
MeN: Mesencephalic nucleus
MoN: Motor nucleus
OS: Oral subnucleus
PAG: Periaquaductal gray
PO: Posterior nucleus
PSN: Principal sensory nucleus
RF: Reticular formation
SN: Spinal nucleus
ST: Spinal tract
TSNC: Trigeminal sensory nucleus complex
VPL: Ventral posterolateral nucleus
VPM: Ventral posteromedial nucleus
V1: Ophthalmic nerve
V2: Maxillary nerve
V3: Mandibular nerve.
